# Clinical outcomes after switching from sulfamethoxazole-trimethoprim to atovaquone due to intolerance in patients with non-HIV *Pneumocystis* pneumonia: a single-center retrospective study

**DOI:** 10.1186/s40780-025-00516-4

**Published:** 2026-01-13

**Authors:** Yukino Shirakawa, Takafumi Nakano, Keisuke Sato, Yuka Takahashi, Mika Higashi, Yukiomi Eguchi, Takuya Yamashina, Tadahiro Ikeuchi, Susumu Kaneshige, Masanobu Uchiyama, Atsushi Togawa, Koichi Matsuo, Hidetoshi Kamimura

**Affiliations:** 1 Department of Pharmacy, Fukuoka University Hospital, 7-45-1 Nanakuma, Jonan-ku, Fukuoka, 814-0180 Japan; 2 Department of Oncology and Infectious Disease Pharmacy, Faculty of Pharmaceutical Sciences, Fukuoka University, 8-19-1 Nanakuma, Jonan-ku, Fukuoka, 814-0180 Japan; 3 Division of Infection Control, Fukuoka University Hospital, 7-45-1 Nanakuma, Jonan-ku, Fukuoka, 814-0180 Japan; 4 Department of Medical Oncology, Hematology, and Infectious Diseases, Faculty of Medicine, Fukuoka University, 7-45-1 Nanakuma, Jonan-ku, Fukuoka, 814-0180 Japan

**Keywords:** *Pneumocystis* pneumonia, Non-HIV infection, Atovaquone, Sulfamethoxazole/trimethoprim

## Abstract

**Background:**

Atovaquone (Atov), a second-line drug, is used to treat patients with *Pneumocystis* pneumonia (PCP) who cannot tolerate sulfamethoxazole/trimethoprim (SMX/TMP). However, the efficacy and safety of Atov are based on clinical trials conducted in patients with human immunodeficiency virus (HIV) or acquired immunodeficiency syndrome, with limited data available on HIV-uninfected individuals with PCP (non-HIV PCP). In this study, we retrospectively evaluated the clinical outcomes of switching from SMX/TMP to Atov in patients with non-HIV PCP.

**Methods:**

The study included patients with non-HIV PCP who were admitted to Fukuoka University Hospital between 2016 and 2023 and initially received SMX/TMP therapy. The primary endpoint was 30-day survival rate from the date of PCP diagnosis. Secondary endpoints included factors associated with mortality and the cumulative incidence of switching from SMX/TMP to Atov.

**Results:**

Of the 56 patients receiving SMX/TMP therapy for PCP, 17 were switched to Atov due to SMX/TMP-related side effects. The Kaplan–Meier estimated 30-day survival was 76.9% in the “remained on” SMX/TMP group and 82.4% in the “switched to” Atov group (log-rank test, *P* = 0.58). Univariable logistic regression analysis of 30-day mortality showed that switching to Atov was not associated with higher mortality compared with continued SMX/TMP therapy (odds ratio 0.71, 95% confidence interval 0.17 to 3.05). The Kaplan–Meier estimated cumulative incidence of switching from SMX/TMP to Atov during the PCP treatment period was 33.8%.

**Conclusion:**

Our data suggest that switching from SMX/TMP to Atov may not be associated with worse survival. Long-term administration of SMX/TMP is often challenging due to its side effects, and in this study, more than 30% of patients were unable to tolerate its therapeutic dose. Our findings support the role of Atov as a viable second-line treatment for PCP in immunocompromised patients, such as those with non-HIV PCP.

## Background

*Pneumocystis* pneumonia (PCP) is a serious respiratory infection that can occur in immunocompromised individuals, especially those infected with human immunodeficiency virus (HIV), and is classified as an acquired immunodeficiency syndrome (AIDS)-defining condition in Japan [1]. In recent years, the incidence of PCP in patients with HIV has declined by approximately 10–20% due to the success of antiretroviral therapy and routine prophylaxis [2, 3]. However, a major emerging concern is the rising incidence of PCP in immunocompromised patients without HIV infection (henceforth termed non-HIV PCP), driven by the extensive utilization of newly developed immunosuppressive drugs for chronic diseases and malignancies [4]. In patients with non-HIV PCP, the disease tends to follow a more acute course, with mortality rates of 30–60%, which are significantly higher than those observed in HIV-PCP [5–7]. Therefore, prompt antimicrobial treatment targeting *Pneumocystis jirovecii* is crucial for patients with suspected PCP. In addition, steroid therapy is often used concomitantly if the patient presents with acute respiratory failure.

Sulfamethoxazole/trimethoprim (SMX/TMP), an antimicrobial agent, is the first-line drug for PCP treatment and is also extensively utilized for PCP prophylaxis. However, SMX/TMP is associated with a high incidence of side effects, complicating adherence to the recommended 14–21-day treatment duration for PCP. Atovaquone (Atov), approved in Japan in 2012, is used for both the prevention and treatment of PCP, and is also used internationally. Compared to SMX/TMP, Atov is expected to have fewer side effects and serve as a useful oral second-line treatment option. However, the therapeutic efficacy and safety data of Atov are primarily derived from clinical trials in patients with HIV/AIDS [8–10], and reports on its therapeutic efficacy in non-HIV PCP are limited. Regarding its preventive use in non-HIV patients, observational studies have been conducted. One study of 480 patients with connective tissue diseases receiving prolonged high-dose glucocorticoids, including 107 patients receiving Atov, found that Atov had a comparable prophylactic effect to SMX/TMP, with fewer adverse events [11]. Another smaller study of 96 patients, including only 7 who received Atov, reported that all patients receiving Atov completed prophylaxis without adverse events, whereas a substantial proportion of those on SMX/TMP discontinued treatment due to side effects [12]. Thus, although evidence on the preventive efficacy of Atov in non-HIV patients is slowly accumulating, data on its therapeutic efficacy remain limited. Considering the distinct clinical course and prognosis of non-HIV PCP compared with HIV PCP [5–7], additional evidence is warranted to evaluate the effectiveness of Atov in treating patients with non-HIV PCP. In the present study, we retrospectively examined the clinical outcomes of switching from SMX/TMP to Atov in patients with non-HIV PCP.

## Methods

### Study design and population

This retrospective, single-center, observational study was approved by the Ethics Committee of Fukuoka University (approval number: H25-02-005). Informed consent was not required due to the retrospective nature of this study. Records of patients admitted to Fukuoka University Hospital between 2016 and 2023 with a diagnosis of PCP were reviewed. Patients who were treated exclusively with oral SMX/TMP or those who were switched from oral SMX/TMP to oral Atov suspension during the PCP treatment period were included in this study. The exclusion criteria were as follows: (i) age < 18 years, (ii) PCP therapy duration < 4 days, (iii) concomitant use of intravenous SMX/TMP or inhaled pentamidine, (iv) missing data, and (v) HIV infection. The included patients were categorized as those who “remained in” the SMX/TMP treatment group (henceforth termed SMX/TMP group) and those who “switched to” the Atov group (henceforth termed Atov group).

### Survey items

We conducted a retrospective evaluation of diagnosis, age, sex, body weight, clinical course, underlying diseases, medical history, and laboratory blood results (including white blood cell count, red blood cell count, platelet count, C-reactive protein, creatinine, aspartate aminotransferase, alanine aminotransferase, lactate dehydrogenase, KL-6, and β-D-glucan) using our electronic medical records system.

### Dosage and criteria for completion of PCP therapy

PCP treatment regimens included oral SMX/TMP, administered at doses according to patient body weight (3600/720 mg or 4800/960 mg per day), or oral Atov suspension (750 mg per dose twice daily). Completion of PCP treatment was determined based on switching to prophylaxis. This indicator was defined according to the dosage and regimen of PCP therapy, such as a reduction in SMX/TMP dose or a change in the Atov regimen from twice daily to once daily. For patients who did not receive prophylaxis after completion of therapy, the end of treatment was determined based on the physicians’ medical records.

### Endpoints and outcome definitions

The primary outcome was 30-day survival from the date of PCP diagnosis (SMX/TMP treatment initiation), reflecting early mortality as a critical indicator of treatment efficacy. The secondary endpoints included 90-day survival, capturing short-term post-treatment outcomes; factors associated with mortality; total treatment duration for PCP; the cumulative incidence of switching from SMX/TMP to Atov; and the selection rate of prophylactic drugs following Atov therapy. The selection of 30- and 90-day survival as endpoints was supported by previous studies evaluating the clinical prognosis of PCP [13–16]. The total treatment duration for PCP was defined as the period from the initiation of a therapeutic dose of SMX/TMP to the completion of treatment with either SMX/TMP or Atov. The reasons for discontinuing SMX/TMP treatment were determined based on the physicians’ medical records.

### Statistical analyses

Data are presented as the median with interquartile range for descriptive statistics, and the median with 95% confidence interval (CI) for Kaplan–Meier estimates. Between-group comparisons were performed using the Mann–Whitney *U*, chi-square, or Fisher’s exact tests. Survival analysis was conducted using Kaplan–Meier survival curves and the log-rank test. For the primary endpoint (30-day survival rate), effect sizes including risk difference (RD) and relative risk (RR) with 95% CIs were calculated; CIs for proportions were estimated using the Wilson score method. As a supplementary sensitivity analysis to address potential immortal time bias in the Atov group, 14-day landmark analyses were performed for 30- and 90-day survival. To explore potential predictors of 30- and 90-day mortality, patients were classified into survivor and non-survivor groups. Univariable logistic regression analysis was performed for each variable to estimate odds ratios (OR) and 95% CI. Due to the limited number of events, multivariable logistic regression was not performed. The treatment duration for PCP was analyzed using Kaplan–Meier curves in the same manner as survival, with deaths treated as censored events. The cumulative incidence of switching from SMX/TMP to Atov during the entire treatment period was analyzed in the same way, with death and treatment completion treated as censored events. All statistical analyses were performed using JMP version 12.0.1 (SAS Institute, Tokyo, Japan). A *P*-value of < 0.05 was considered statistically significant.

## Results

### Patient demographics

**Fig. 1:**
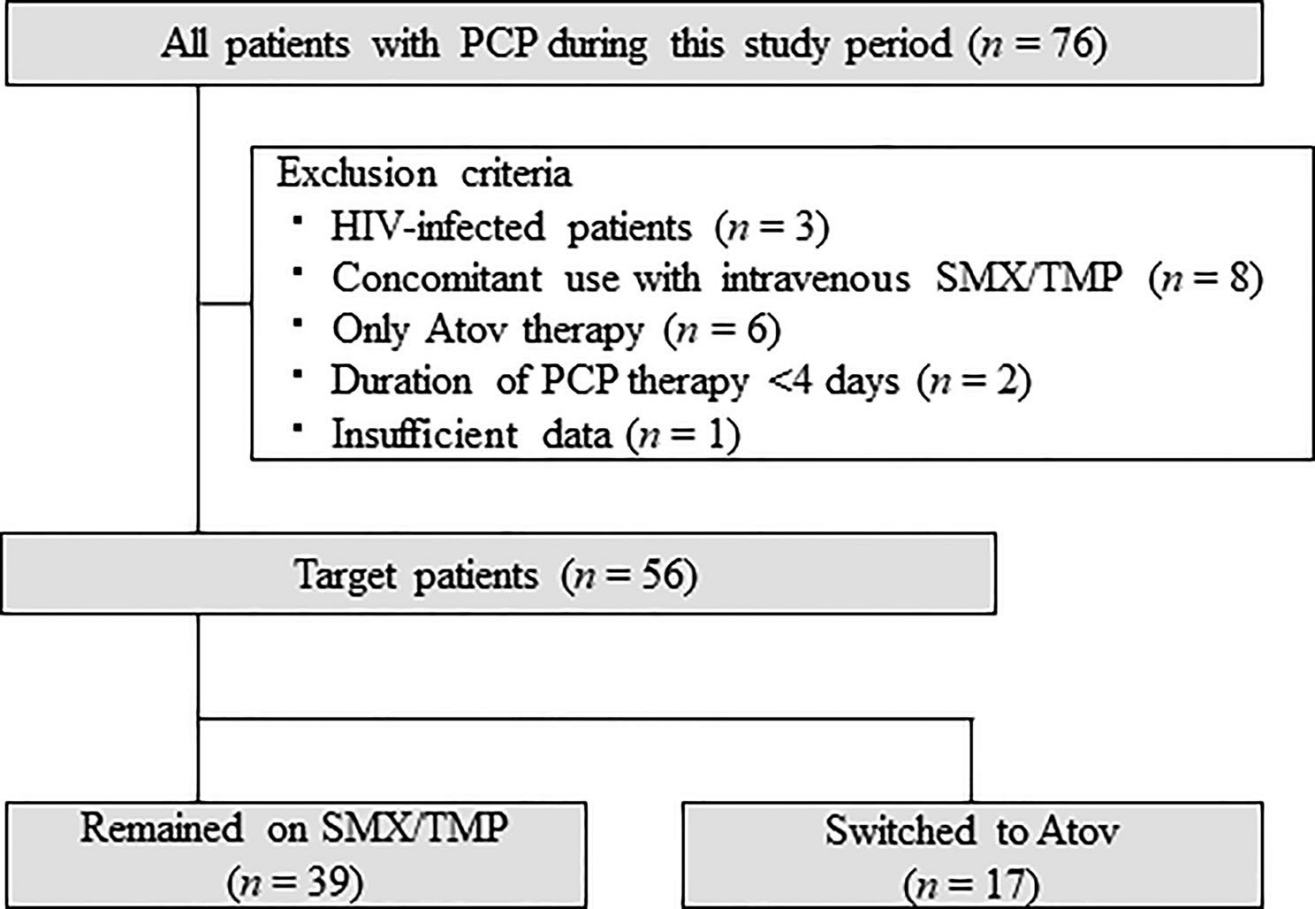
Flowchart of patient selection for this study. Atov, atovaquone; HIV, human immunodeficiency virus; PCP, *Pneumocystis* pneumonia; SMX/TMP, sulfamethoxazole/trimethoprim

A flowchart of the study is shown in Fig. 1. Among the total 76 patients diagnosed with PCP who were enrolled in this study, 56 were included, comprising those who remained on SMX/TMP therapy (*n* = 39) and those who switched to the Atov therapy (*n* = 17). In the Atov group, all patients received Atov therapy for at least one week after switching from SMX/TMP. Moreover, no patient had a history of allergy to PCP prophylactic or therapeutic drugs before treatment initiation, and none were treated with intravenous pentamidine during the study period.

Table 1 summarizes the baseline clinical and demographic characteristics of the 56 patients at the time of PCP treatment initiation. Most participants were immunocompromised patients with underlying hematological malignancies (33.9%), solid tumors (30.4%), or collagen disorders (19.6%), such as systemic lupus erythematosus or rheumatoid arthritis. Other patients (16.1%) included those receiving oral steroid treatment for conditions such as ulcerative colitis, adult-onset Still’s disease, and microscopic polyangiitis. Approximately 95% of the patients were not receiving prophylactic therapy for PCP.

**Table 1:** Characteristics of the patients included in this study

Variables	Overall (*n* = 56)	Group		*P*-value
		Remained on SMX/TMP (*n* = 39)	Switched to Atov (*n* = 17)	
***Characteristics***				
Age, years	68.0 (57.8–74.8)	67.0 (57.0–73.0)	69.0 (64.5–80.0)	0.24
Men, *n* (%)	31 (55.4)	21 (53.8)	10 (58.8)	0.73
Body weight, kg	49.6 (45.6–59.9)	48.9 (45.7–60.0)	50.7 (45.4–59.2)	0.89
***Underlying disease***				0.28
HM, *n* (%)	19 (33.9)	14 (35.9)	5 (29.4)	
Solid tumors, *n* (%)	17 (30.4)	13 (33.3)	4 (23.5)	
Collagen disorder, *n* (%)	11 (19.6)	5 (12.8)	6 (35.3)	
Others, *n* (%)	9 (16.1)	7 (17.9)	2 (11.8)	
***Immunosuppressive medications***				
Anticancer drugs^*^, *n* (%)	27 (48.2)	20 (51.3)	7 (41.2)	0.49
Corticosteroids, *n* (%)	26 (46.4)	16 (41.0)	10 (58.8)	0.22
Methotrexate, *n* (%)	10 (17.9)	5 (12.8)	5 (29.4)	0.27
***Prophylaxis***				0.70
SMX/TMP, *n* (%)	2 (3.6)	1 (2.6)	1 (5.9)	
Atov, *n* (%)	1 (1.8)	1 (2.6)	0 (0)	
Nothing, *n* (%)	53 (94.6)	37 (94.9)	16 (94.1)	
***Laboratory data***				
WBC, ×10^3^/µL	8.1 (5.7–12.1)	9.8 (6–13.6)	6.7 (5.0–10.0)	0.10
RBC, ×10^6^/µL	3.1 (2.6–3.8)	3.0 (2.6–3.7)	3.5 (2.9–4.0)	0.15
PLT, ×10^3^/µL	240.5 (127.5–331.3)	248.0 (100.0–329.0)	240.0 (168.5–334.5)	0.74
CRP, mg/dL	8.6 (3.9–13.8)	8.7 (3.9–12.7)	7.7 (4.5–14.5)	0.95
Cr, mg/dL	0.7 (0.62–0.83)	0.7 (0.6–0.8)	0.8 (0.6–0.9)	0.07
AST, U/L	31.0 (23.3–42.3)	31.0 (25.0–43.0)	27.0 (18.5–45.5)	0.29
ALT, U/L	28.0 (15.3–28.0)	30.0 (16.0–42.0)	19.0 (9.0–34.0)	0.67
LDH, U/L	345.5 (263.4–499.0)	357.0 (273.0–493.0)	329.0 (240.0–501.5)	0.56
KL-6, U/mL	456.5 (314.8–1032.8)	402.0 (323.0–689.0)	620.0 (304.0–1375.0)	0.28
β-d-glucan, pg/mL	35.5 (14.3–93.3)	31.4 (14.0–144.4)	37.7 (14.5–92.6)	0.55
***Concomitant therapy*** ***for PCP***				
PSL ≥ 1 mg/kg/day, *n* (%)	53 (94.6)	36 (92.3)	17 (100.0)	0.60

^*^Received within 3 months before PCP onset. Values are presented as number (%) or median (interquartile range)

ALT, alanine aminotransferase; AST, aspartate aminotransferase; Atov, atovaquone; Cr, creatinine; CRP, C-reactive protein; KL-6, Krebs von den Lungen-6; LDH, lactate dehydrogenase; MTX, methotrexate; HM, hematological malignancies; PCP, *Pneumocystis* pneumonia; PLT, platelet; PSL, prednisolone; RBC, red blood cell; SMX/TMP, sulfamethoxazole/trimethoprim; WBC, white blood cell

The background characteristics of the patients in both groups are presented in Table 1. Age, sex, body weight, underlying diseases, PCP prophylactic therapy, and laboratory data were not significantly different between the two groups. Additionally, most patients in both groups received prednisolone (PSL) therapy (≥ 1 mg/kg/day) for PCP-induced respiratory failure.

### Survival and treatment period

**Fig. 2:**
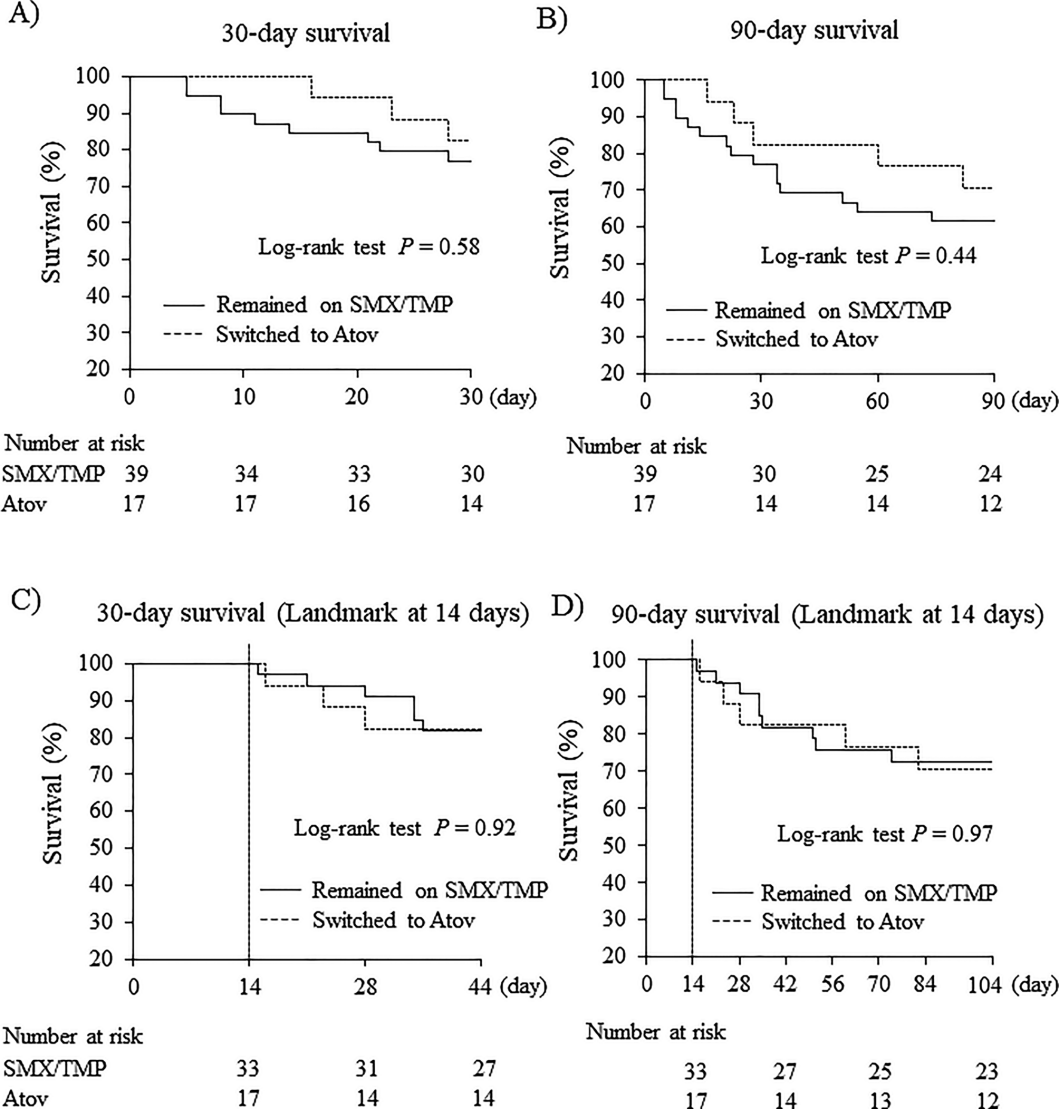
Kaplan–Meier 30- and 90-day survival curves, with landmark analyses at 14 days, for SMX/TMP and Atov groups. Figures indicate 30-day survival (**A**), 90-day survival (**B**), 30-day survival with a 14-day landmark analysis (**C**), and 90-day survival with a 14-day landmark analysis (**D**). Survival probabilities were compared between groups using the log-rank test. Atov, atovaquone; SMX/TMP, sulfamethoxazole/trimethoprim

The Kaplan–Meier survival curves are shown in Fig. 2. The Kaplan–Meier estimated 30-day survival rate, the primary endpoint, was 76.9% in the SMX/TMP group and 82.4% in the Atov group, with no significant difference between the groups (log-rank test, *P* = 0.58; Fig. 2A). The RD was 0.06 (95% CI -0.17 to 0.28), and the RR was 1.07 (95% CI 0.81 to 1.41), indicating no substantial difference between groups. Similarly, the Kaplan–Meier estimated 90-day survival rate was 61.5% in the SMX/TMP group and 70.6% in the Atov group, with no significant difference between groups (log-rank test, *P* = 0.44; Fig. 2B). As a supplementary analysis, the the Kaplan–Meier estimated 30-day survival rate after the 14-day landmark analysis was 81.8% in the SMX/TMP group and 82.4% in the Atov group, with no significant difference between the groups (log-rank test, *P* = 0.92; Fig. 2C). The Kaplan–Meier estimated 90-day survival rate after the 14-day landmark analysis was 70.6% in the SMX/TMP group and 69.7% in the Atov group, with no significant difference (log-rank test, *P* = 0.97; Fig. 2D). No cases of PCP recurrence were observed in either group during the 90-day follow-up period. Univariable logistic regression analysis was performed to evaluate potential predictors of 30- and 90-day mortality. Switching to Atov was not associated with mortality at either time point compared with continued SMX/TMP therapy (30-day OR 0.71, 95% CI 0.17 to 3.05; 90-day OR 0.80, 95% CI 0.20 to 3.20). No other factors showed a significant association with mortality (Tables 2 and 3).

**Table 2:** Univariable analysis of potential predictors of 30-day mortality in the overall cohort

Variable	Survivors (*n* = 44)	Non-survivors (*n* = 12)	OR	95% CI	*P*-value
***Characteristics***					
Age, years	68 (56–74)	69 (62–78)	1.03	0.98–1.10	0.20
Male, *n* (%)	26 (59.1)	5 (41.7)	0.49	0.14–1.80	0.34
Body weight, kg	50.1 (45.4–58.6)	48.7 (46.6–64.6)	1.01	0.96–1.06	0.79
***Underlying disease***					
Cancer, *n* (%)	30 (68.2)	6 (50.0)	0.47	0.13–1.71	0.31
Collagen disorder, *n* (%)	8 (18.2)	3 (25.0)	1.50	0.33–6.82	0.69
***Immunosuppressive medications***					
Anticancer drugs^*^, *n* (%)	22 (50.0)	5 (41.7)	0.71	0.20–2.60	0.75
Corticosteroids, *n* (%)	18 (40.9)	8 (66.7)	2.89	0.75–11.1	0.19
Methotrexate, *n* (%)	7 (15.9)	3 (25.0)	1.76	0.38–8.19	0.43
***Treatment for PCP***					
PSL ≥ 1 mg/kg/day, *n* (%)	42 (95.5)	11 (91.7)	0.52	0.10–6.32	0.52
Switched to Atov, *n* (%)	14 (31.8)	3 (25.0)	0.71	0.17–3.05	0.74

^*^Received within 3 months before PCP onset. Values are presented as number (%) or median (interquartile range)

Atov, atovaquone; CI, confidence interval; OR, odds ratio; PCP, *Pneumocystis* pneumonia;

PSL, prednisolone

**Table 3:** Univariable analysis of potential predictors of 90-day mortality in the overall cohort

Variable	Survivors (*n* = 36)	Non-survivors (*n* = 20)	OR	95% CI	*P*-value
***Characteristics***					
Age, years	69 (56–76)	68 (61–71)	1.01	0.97–1.05	0.70
Male, *n* (%)	20 (55.6)	11 (55.0)	0.98	0.33–2.94	1.00
Body weight, kg	48.9 (45.3–58.6)	50.1 (47.1–63.0)	1.00	0.96–1.05	0.85
***Underlying disease***					
Cancer, *n* (%)	24 (66.7)	12 (60.0)	0.75	0.24–2.33	0.77
Collagen disorder, *n* (%)	8 (22.2)	3 (15.0)	0.62	0.14–2.65	0.73
***Immunosuppressive medications***					
Anticancer drugs^*^, *n* (%)	16 (44.4)	11 (55.0)	1.53	0.51–4.59	0.58
Corticosteroids, *n* (%)	16 (44.4)	10 (50.0)	1.25	0.42–3.74	0.78
Methotrexate, *n* (%)	6 (16.7)	4 (20.0)	1.25	0.31–5.08	0.73
***Treatment for PCP***					
PSL ≥ 1 mg/kg/day, *n* (%)	35 (97.2)	18 (90.0)	0.26	0.08–3.03	0.29
Switched to Atov, *n* (%)	12 (33.3)	5 (25.0)	0.67	0.20–2.27	0.56

^*^Received within 3 months before PCP onset. Values are presented as number (%) or median (interquartile range)

Atov, atovaquone; CI, confidence interval; OR, odds ratio; PCP, *Pneumocystis* pneumonia;

PSL, prednisolone

**Fig. 3:**
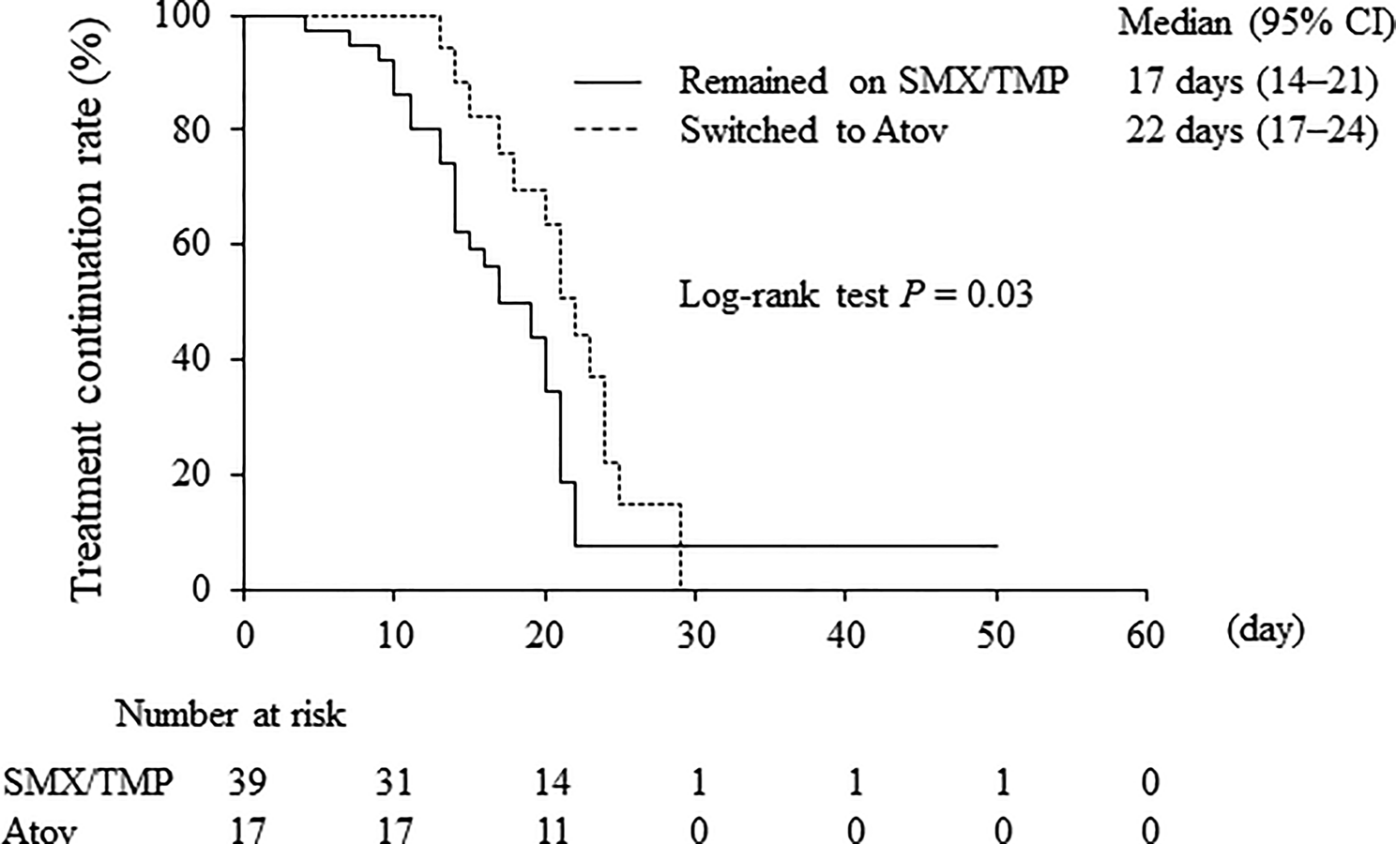
Kaplan–Meier comparison of total treatment duration for PCP. Atov, atovaquone; CI, confidence interval; PCP, *Pneumocystis* pneumonia; SMX/TMP, sulfamethoxazole/trimethoprim

Among patients who showed treatment efficacy, Kaplan–Meier analysis estimated a median total treatment duration for PCP of 17 days (95% CI 14 to 21) in the SMX/TMP group and 22 days (95% CI 17 to 24) in the Atov group (Fig. 3). The total treatment duration was significantly longer in the Atov group than in the SMX/TMP group (log-rank test, *P* = 0.03). In the Atov group, the median duration of SMX/TMP administration was 7 days (95% CI 6 to 9) and the median duration of Atov administration was 12 days (95% CI 10 to 18), both calculated using the Kaplan–Meier method.

### Factors and outcomes of SMX/TMP discontinuation

The Kaplan–Meier estimated cumulative incidence of switching from SMX/TMP to Atov due to adverse events during PCP treatment was 33.8%. In all 17 patients who experienced adverse events, the events occurred within 2 weeks of starting SMX/TMP treatment and they were switched to Atov: 7 patients (41.2%) were switched to Atov on days 4–7, and 10 patients (58.8%) were switched to Atov on days 8–14. The details of the reasons for switching from SMX/TMP to Atov were as follows. The major reasons included renal dysfunction (41.2%, 7/17 cases), loss of appetite due to nausea (17.6%, 3/17 cases), and myelosuppression such as thrombocytopenia and agranulocytosis (17.6%, 3/17 cases). Other reasons were eruption (11.8%, 2/17 cases), hepatic dysfunction (5.9%, 1/17 cases), and QT prolongation (5.9%, 1/17 cases). No cases of severe PCP were attributed to SMX-/TMP-resistant *P. jirovecii*. Among the 33 patients who completed SMX/TMP therapy, all continued to receive SMX/TMP as prophylaxis. Moreover, of the 15 patients who completed Atov therapy, 53.3% (8/15 cases) remained on Atov for prophylaxis, 33.3% (5/15 cases) switched back to SMX/TMP, and 13.3% (2/15 cases) did not receive any prophylaxis.

## Discussion

PCP is an emerging threat to non-HIV immunocompromised patients, especially those receiving immunosuppressive or anticancer drugs. In patients with non-HIV PCP, the disease can progress rapidly and cause severe respiratory failure with poor prognosis [17]. This is due to several factors including delayed diagnosis, lack of prophylaxis, advanced age, and comorbidities [6, 18]. In Japan, PCP treatment is typically initiated with SMX/TMP. Patients who cannot tolerate SMX/TMP due to side effects or allergies are switched to Atov. However, the clinical prognosis of patients with non-HIV PCP following a switch to Atov has not been fully investigated. This study evaluated the effects of switching from SMX/TMP to Atov on the clinical outcomes in patients with non-HIV PCP. Our results showed that while patients who switched to Atov had a longer total duration for PCP treatment than those who remained on SMX/TMP; however, their 30-day survival was not significantly affected. These findings suggest that transitioning to Atov therapy may not be associated with substantially worse 30-day survival, indicating that it could be a clinically reasonable alternative for patients who are intolerant to SMX/TMP therapy. This is the first report to compare the clinical outcomes, including 30-day survival, between patients with non-HIV PCP who remained on SMX/TMP and those who switched to Atov. Considering the limited data on Atov’s efficacy in patients with non-HIV PCP, our study offers valuable insights that contribute to the advancement of PCP treatment strategies.

In this study, the 30-day survival rate was 76.9% in the SMX/TMP group. Several studies have examined the effect of SMX/TMP on survival in patients with non-HIV PCP [19–21], showing that the 30-day survival with conventional-dose SMX/TMP (TMP 15–20 mg/kg/day) ranges from 79.0 to 85.0% [20, 21]. Therefore, our results are consistent with these previous findings. We then compared survival between the SMX/TMP and Atov groups and found that switching to Atov did not affect the 30-day survival. Importantly, SMX/TMP was discontinued due to either its side effects or allergic reactions. However, these adverse effects resolved in all patients after switching to Atov. Although Atov is known to be associated with side effects, such as skin eruptions, cytopenia, and liver dysfunction [22], no patient in this study discontinued Atov due to adverse events. These results suggest that compared with therapeutic-dose SMX/TMP, Atov has a lower incidence of side effects, suggesting that it can be a viable option as a second-line drug for PCP. In contrast, the 90-day survival rate was slightly lower in the SMX/TMP group than in the Atov group. However, drug switching, cancer, and age were not independent predictors of increased 30-day mortality. Most patients who died within 31–90 days following PCP onset had already completed their PCP treatment regimen, and there were no cases of PCP recurrence. Thus, in this study, we posited that the influence of various factors, including underlying diseases, on overall prognosis may have enhanced with the extension of the observation period, along with the deterioration of the overall condition triggered by PCP.

The Atov group had a longer PCP treatment duration than the SMX/TMP group, which may be due to various factors. Differences in PCP severity among the groups may have influenced treatment duration. In patients infected with HIV, the therapeutic efficacy of Atov for severe PCP is reported to be lower than that of SMX/TMP [9]. Therefore, Atov is recommended for mild to moderate PCP. In this study, due to lack of clinical information, we could not directly compare the degree of acute respiratory failure, a crucial indicator of severity. Instead, we assessed the relative incidence of acute respiratory failure due to PCP by examining the proportion of PSL use, and found no difference between the two groups. In addition, the exclusion of patients who received intravenous SMX/TMP may have potentially minimized severity differences. Despite these efforts to control the confounding factors related to severity, this was a retrospective observational study; therefore, unmeasured differences, such as variations in respiratory status and bacterial load, may still exist. Another possible explanation for the prolonged treatment duration in the Atov group might be the cautious approach after switching, due to the occurrence of adverse events during SMX/TMP therapy. The Japanese Infectious Disease Treatment Guidelines recommend 14 days of SMX/TMP therapy for non-HIV PCP and 21 days for HIV-infected PCP [23]. Therefore, the occurrence of adverse events and the subsequent use of second-line drugs may have led to longer treatment durations, aligning with the upper limit of the recommended treatment period. Further studies are warranted to determine the optimal treatment duration after switching to Atov therapy in patients with non-HIV PCP.

In this study, few patients received prophylactic treatment before the onset of PCP, despite the recommendation that prophylaxis with SMX/TMP be initiated when patients are receiving treatment with PSL at ≥ 20 mg/day for at least 4 weeks [24]. The conventional prophylactic dose of SMX/TMP in patients with normal kidney function is typically either two tablets (800/160 mg) per day, three times per week, or one tablet (400/80 mg) daily [25]. In this study, patients who completed SMX/TMP therapy continued to receive SMX/TMP as prophylaxis. Notably, approximately 30% (5/17) of those who completed Atov therapy returned to SMX/TMP for prophylaxis, being initiated at a lower than recommended SMX/TMP dose. The reason for returning to SMX/TPM or forgoing prophylactic treatment altogether was the economic disadvantage of Atov. Preventive treatment with SMX/TMP is achieved by reducing the dose to a level that minimizes side effects and allows most patients to tolerate it. Consequently, in this study, only one patient was unable to continue receiving SMX/TMP, even at a reduced dose. These findings underscore the need for further research on prophylactic strategies for patients who experience adverse events with therapeutic doses of SMX/TMP, especially immunocompromised individuals.

Our study has some limitations. First, it was a retrospective, single-center, observational study with a limited sample size. In particular, the Atov group included only 17 patients, which limited statistical power. Therefore, the absence of a significant difference in survival between the SMX/TMP and Atov groups should not be interpreted as evidence of equivalence. Furthermore, multiple confounding factors may have influenced the outcomes, including patients’ underlying diseases, comorbidities, infection severity, use of concomitant medications, and multiple infections. Although a multivariable analysis would have been preferable to adjust for these factors, the small sample size made this approach unfeasible. Consequently, we were only able to perform univariable logistic regression analyses, which should be interpreted as exploratory. Second, the duration of SMX/TMP therapy in patients who switched to Atov varied, which may have influenced both survival outcomes and total treatment duration. Because of the limited number of cases, we were unable to formally evaluate the impact of the timing of switching. To partially account for potential immortal time bias, survival rates were evaluated using a 14-day landmark analysis; however, these effects could not be fully eliminated. Third, the total treatment period should have ideally been evaluated based on symptom resolution and bacterial load reduction rather than the number of days of drug treatment. However, clinical data regarding overall patient conditions, such as respiratory status and bacterial clearance by polymerase chain reaction (PCR) were not available. Similarly, not all patients were diagnosed based on confirmatory tests such as PCR at the time of diagnosis; rather, the diagnosis was primarily based on clinical symptoms and patient background. Therefore, diagnostic certainty may not have been uniform across all cases. These limitations should be considered when interpreting our results, and future studies should incorporate these parameters along with more robust diagnostic confirmation. Fourth, there remains concern about possible differences in baseline PCP severity between the two groups. A comprehensive assessment of baseline PCP severity ideally requires evaluation of oxygenation indices, clinical respiratory status, severity scores (such as Acute Physiologic Assessment and Chronic Health Evaluation II or Sequential Organ Failure Assessment scores), imaging findings, and laboratory data. However, because this was a retrospective study, not all of these data were available, and imaging findings could not be systematically analyzed. The most consistently available parameter was arterial oxygen partial pressure at the time of diagnosis (PaO2), although it was not obtained for all patients. Even when these data were available, interpretation of PaO₂ results was difficult because fraction of inspired oxygen (FiO₂) at the time of arterial blood gas sampling was often unknown for many patients due to variations in oxygen delivery devices and flow rates. Therefore, we only analyzed patients with available PaO2 to FiO₂ ratio data, and no significant difference was observed between the groups. However, assessment based solely on the available data does not fully reflect baseline disease severity. We excluded patients with clearly severe PCP who received intravenous SMX/TMP treatment; however, ideally, baseline severity should have been assessed in all patients. As a result, a comprehensive comparison of PCP severity between the two groups was not possible. These limitations should be taken into account when interpreting the study findings, including the primary outcome of 30-day survival. To overcome these limitations, multicenter prospective observational studies are necessary.

In conclusion, this retrospective, single-center study does not allow for definitive conclusions, but provides exploratory insights into the treatment of non-HIV PCP. Our data suggest that switching from SMX/TMP to Atov may not be associated with worse survival. Considering that therapeutic doses of SMX/TMP are often difficult to maintain due to frequent adverse effects (> 30% of patients in our cohort), Atov has the potential to be a clinically reasonable alternative in cases of SMX/TMP intolerance among patients with non-HIV PCP. Further prospective studies are warranted to confirm these observations.

## Data Availability

Not applicable.

## Abbreviations

AIDS: Acquired immunodeficiency syndrome
Atov: Atovaquone
FiO₂: Fraction of inspired oxygen
HIV: Human immunodeficiency virus
KL-6: Krebs von den lungen-6
PaO₂: Arterial oxygen partial pressure
PCP: Pneumocystis pneumonia
PCR: Polymerase chain reaction
SMX/TMP: Sulfamethoxazole/trimethoprim
